# Trends in the Use of Neoadjuvant Systemic Therapy for Head and Neck Squamous Cell Carcinoma

**DOI:** 10.1001/jamanetworkopen.2025.39778

**Published:** 2025-10-28

**Authors:** Wesley L. Cai, Vanessa Helou, Katie M. Carlson, Angela L. Mazul, Dan P. Zandberg, Matthew E. Spector, Jose P. Zevallos, Robert L. Ferris, Kevin J. Contrera

**Affiliations:** 1 Department of Otolaryngology–Head and Neck Surgery, University of Pittsburgh Medical Center, Pittsburgh, Pennsylvania; 2 Cancer Epidemiology and Prevention Program, UPMC Hillman Cancer Center, University of Pittsburgh, Pittsburgh, Pennsylvania; 3 UPMC Hillman Cancer Center, University of Pittsburgh, Pittsburgh, Pennsylvania; 4 UNC Lineberger Comprehensive Cancer Center, University of North Carolina, Chapel Hill

## Abstract

**Importance:**

A paradigm shift is currently under way for the treatment of head and neck squamous cell carcinoma (HNSCC). Recent trials have demonstrated potential effectiveness of neoadjuvant systemic therapy (NST), including chemotherapy and immunotherapy; however, data on application of this approach are limited.

**Objective:**

To evaluate trends in the use of NST in patients with HNSCC from 2004 to 2022 and factors associated with treatment.

**Design, Setting, and Participants:**

This multicenter, retrospective cohort study used the National Cancer Database to identify 312 748 patients diagnosed with HNSCC from January 1, 2004, to December 31, 2022, who received definitive surgery. NST was defined as therapy administered at least 8 weeks before definitive surgery.

**Exposures:**

Sociodemographic factors and clinical characteristics.

**Main Outcomes and Measures:**

Adjusted risk of receiving neoadjuvant immunotherapy and chemotherapy across the analyzed years.

**Results:**

Among 312 748 patients (mean [SD] age, 63.3 [12.2] years; 218 218 [69.8%] male) who underwent surgery between 2004 and 2022, 1989 (0.6%) received NST, and among these, 1372 (69.0%) received neoadjuvant chemotherapy, 726 (36.5%) received neoadjuvant immunotherapy, and 109 (5.5%) received both. The first year of recorded neoadjuvant immunotherapy was 2007, with a use rate of 0.02%. Use began to increase in 2013 with a rate of 0.14%, peaking at 0.73% in 2019 but decreasing to 0.27% in 2022. From 2007 to 2022, the adjusted risk of receiving neoadjuvant immunotherapy increased by 22.5% per year (risk ratio [RR], 1.22; 95% CI, 1.19-1.26), whereas the adjusted risk of receiving chemotherapy decreased by −2.5% per year (RR, 0.97; 95% CI, 0.96-0.99). Sites with the largest increases in neoadjuvant immunotherapy use since 2013 were the hypopharynx (from 0.25% to 1.30%), gums and other oral cavity (from 0.19% to 0.58%), and tongue (from 0.18% to 0.27%). Patients who received neoadjuvant immunotherapy were more likely to have private insurance (342 [47.1%] vs 125 542 [40.2%]; *P* < .001), more likely to have stage IV disease (394 [54.3%] vs 100 565 [32.2%]; *P* < .001), and less likely to identify as Black (33 [4.5%] vs 21 384 [6.9%]; *P*  = .01).

**Conclusions and Relevance:**

In this retrospective cohort study of HNSCC, rates of neoadjuvant immunotherapy nearly doubled between 2013 and 2022, whereas neoadjuvant chemotherapy use significantly decreased from 2007 to 2022. These trends highlight the evolving therapeutic landscape for HNSCC and provide context for emerging data on neoadjuvant therapy.

## Key Points

**Question:**

What are the trends in neoadjuvant systemic therapy use in head and neck squamous cell carcinoma (HNSCC)?

**Findings:**

In this cohort study of 312 748 patients with HNSCC undergoing definitive surgery from 2004 to 2022, 0.6% received neoadjuvant systemic therapy. Neoadjuvant immunotherapy use significantly increased over time, whereas neoadjuvant chemotherapy use decreased, and neoadjuvant immunotherapy recipients were more likely to have private insurance and stage IV disease and less likely to be of Black race.

**Meaning:**

Findings from this study suggest that increasing neoadjuvant immunotherapy use reflects evolving treatment practices in HNSCC and highlights disparities, underscoring the need to align emerging therapies with health care access and disease severity.

## Teaser

This cohort study of patients with head and neck squamous cell carcinoma evaluates trends in neoadjuvant systemic therapy use and explores associations with sociodemographic and clinical characteristics.

## Introduction

Head and neck squamous cell carcinoma (HNSCC) represents a substantial global health burden, accounting for approximately 4% of all cancers worldwide.^1^ Despite treatment advances, survival rates for patients with HNSCC have been poor, highlighting the need for more effective therapeutic strategies.^2^ One treatment approach is neoadjuvant systemic therapy (NST), which includes chemotherapy and immunotherapy, administered before definitive surgery. Traditionally, neoadjuvant chemotherapy has been used to reduce distant metastases, improve organ preservation, and personalize treatment.^3^ Although promising, trials using neoadjuvant chemotherapy have failed to demonstrate significantly improved survival compared with upfront surgery.^3^

The introduction of immune checkpoint inhibitors (ICIs), particularly antiprogrammed cell death 1 agents, has reshaped the therapeutic landscape of HNSCC. These agents are approved by the US Food and Drug Administration for recurrent or metastatic HNSCC.^4,5,6^ More recently, the phase 3 KEYNOTE-689 trial demonstrated that neoadjuvant and adjuvant pembrolizumab significantly improved event-free survival in patients with locally advanced HNSCC.^7^ On this basis, pembrolizumab has been approved for the first-line treatment for patients with resectable, locally advanced HNSCC whose tumor expressed programmed cell death 1 ligand 1, signaling a paradigm shift in standard of care. However, robust data on NST use in HNSCC remain limited. To address this gap, we use data from the National Cancer Database (NCDB) to evaluate trends in the use of NST for HNSCC between 2004 and 2022 and explored their associations with sociodemographic and clinical characteristics.

## Methods

### Population

The NCDB was queried to identify patients diagnosed with HNSCC from January 1, 2004, through December 31, 2022, reported across multiple facilities. Eligibility criteria included patients who underwent definitive surgery and whose tumor matched the following organ sites and histology. Nine organ sites, defined by the *International Classification of Diseases for Oncology, Third Revision (ICD-O-3)* codes, were selected: lip (C000-C009), tongue (C019-C029), floor of mouth (C040-C049), gum and other mouth (C030-C039, C050-C059, C060-C069), tonsil (C090-C099), oropharynx (C100-C109), pharynx (C140, C142, C148), hypopharynx (C129, C130-C139), and larynx (C320-C329). Squamous cell carcinoma was identified by histology codes 8070 to 8076, 8078, 8084 to 8086, and 8560. NST was defined as either chemotherapy or immunotherapy administered 8 weeks or less prior to definitive surgery. This cutoff was determined based on the observation that most clinical trials of neoadjuvant immunotherapy used a neoadjuvant window of up to 8 weeks.^8^ Notably, similar results were obtained when 3 weeks was used as the cutoff. This study was determined to be exempt from review and informed consent by the University of Pittsburgh Institutional Review Board because NCDB uses deidentified, retrospective data. This study followed the Strengthening the Reporting of Observational Studies in Epidemiology (STROBE) reporting guideline for cohort studies.

Participant race and ethnicity were classified based on the NCDB data, which included the following categories: Asian, Black, White, and other. Asian race was defined as Chinese, Japanese, Filipino, Korean, Vietnamese, Thai, Laotian, Hmong, Asian Indian, and other Asian. Other was used to encompass all ethnicities annotated in the NCDB that did not fall within Asian, Black, and White. Data on race and ethnicity were collected to evaluate potential disparities in outcomes and to ensure that study findings are applicable across diverse patient populations.

### Statistical Analysis

All statistical analyses were performed using R, version 4.4.0 (R Project for Statistical Computing). Descriptive statistics of sociodemographic and clinical variables stratified by therapy type were performed using χ^2^ testing. For nonmonotonic trend analysis, χ^2^ test of independence was used to test for deviation from null. For monotonic trends, significance was tested with the Mann-Kendall test using frequency data and the Cochran-Armitage test with proportion data. To obtain risk ratios (RRs), mixed-effects Poisson regression was used with facility identification as the clustering variable. Fixed variables were year of diagnosis, age, sex, race, insurance status, overall stage, urban or rural context of residence, zip code median income, and Charlson-Deyo comorbidity score. Independence of effects was assessed with variance inflation factors. Patients with missing variable data were excluded from analyses. A 2-sided *P* < .05 was considered statistically significant.

## Results

Of 312 748 unique patients with HNSCC diagnosed from 2004 to 2022 who underwent definitive surgery (mean [SD] age, 63.3 [12.2] years; 218 218 [69.8%] male and 94 530 [30.2%] female; 7805 [2.5%] Asian, 21 417 [6.8%] Black, 276 798 [88.5%] White, and 3528 [1.1%] other), 213 460 (68.3%) had oral cavity cancer, 66 680 (21.3%) had pharyngeal cancer, and 66 397 (21.2%) had laryngeal cancer. A total of 1989 (0.6%) received NST, and among these, 1372 (69.0%) received neoadjuvant chemotherapy, 726 (36.5%) received neoadjuvant immunotherapy, and 109 (5.5%) received both. Of patients who received neoadjuvant immunotherapy, 551 (75.9%) had oral cavity cancer, 129 (17.8%) had pharyngeal cancer, and 67 (9.2%) had laryngeal cancer.

Receipt of neoadjuvant immunotherapy differed significantly across all sociodemographic and clinical characteristics except Charlson-Deyo comorbidity score and p16 status (Table). Compared with patients who did not receive neoadjuvant immunotherapy, those who did receive neoadjuvant immunotherapy were more likely to be treated at academic centers (554 [76.3%] vs 162 221 [52.0%]; *P* < .001), live in metropolitan areas with populations between 250 000 and 1 million (578 [79.6%] vs 239 741 [76.8%], *P* < .001), live in neighborhoods with the highest median income (258 [35.5%] vs 95 093 [30.5%]; *P* < .001), have private insurance (342 [47.1%] vs 125 542 [40.2%]; *P* < .001), and have stage IV disease (394 [54.3%] vs 100 565 [32.2%]; *P* < .001) and less likely to identify as Black (33 [4.5%] vs 21 384 [6.9%]; *P*  = .01). Within recipients of neoadjuvant immunotherapy, those who lived in metropolitan areas compared with urban or rural areas did not differ in disease stage or insurance status.

**Table.** Sociodemographic and Clinical Characteristics of Patients With Head and Neck Squamous Cell Carcinoma Stratified by Receipt of Neoadjuvant Immunotherapy

Characteristic	No. (%) of patients^a^		*P* values	
	Received neoadjuvant immunotherapy (n = 726)	Did not receive neoadjuvant immunotherapy (n = 312 022)	Individual level^b^	All levels^c^
Treatment category				
Neoadjuvant immunotherapy only	617 (85.0)	NA	NA	NA
Neoadjuvant immunotherapy and chemotherapy	109 (15.0)	NA	NA	NA
Year of diagnosis				
2007	2 (0.3)	13 187 (4.2)	NA	NA
2008	3 (0.4)	13 529 (4.3)	NA	NA
2009	1 (0.1)	13 783 (4.4)	NA	NA
2010	0	14 662 (4.7)	NA	NA
2011	4 (0.6)	15 534 (5.0)	NA	NA
2012	0	15 796 (5.1)	NA	NA
2013	23 (3.2)	17 000 (5.4)	NA	NA
2014	17 (2.3)	17 300 (5.5)	NA	NA
2015	33 (4.5)	17 899 (5.7)	NA	NA
2016	45 (6.2)	18 828 (6.0)	NA	NA
2017	94 (12.9)	19 270 (6.2)	NA	NA
2018	119 (16.4)	20 050 (6.4)	NA	NA
2019	150 (20.7)	20 427 (6.5)	NA	NA
2020	105 (14.5)	19 158 (6.1)	NA	NA
2021	79 (10.9)	20 498 (6.6)	NA	NA
2022	51 (7.0)	18 824 (6.0)	NA	NA
Age, y				
Mean (SD)	60.7 (11.7)	63.3 (12.2)	NA	NA
Median (range)	62 (54.0-68.0)	63 (55.0-72.0)	NA	NA
Area of residence^d^				
Metropolitan	578 (79.6)	239 741 (76.8)	NA	<.001
Urban	97 (13.4)	49 880 (16.0)	NA	
Rural	12 (1.7)	6795 (2.2)	NA	
Missing data	39 (5.4)	15 606 (5.0)	NA	
Race				
Asian^e^	24 (3.3)	7781 (2.5)	.21	.01
Black	33 (4.5)	21 384 (6.9)	.01	
White	656 (90.4)	276 142 (88.5)	.28	
Other^f^	10 (1.4)	2534 (0.8)	.14	
Missing data	3 (0.4)	3197 (1.0)	NA	
Facility location				
Middle Atlantic	236 (32.5)	43 576 (14.0)	NA	<.001
South Atlantic	91 (12.5)	64 852 (20.8)	NA	
West North Central	84 (11.6)	30 201 (9.7)	NA	
Pacific	76 (10.5)	31 928 (10.2)	NA	
East North Central	72 (9.9)	55 133 (17.7)	NA	
New England	57 (7.9)	16 057 (5.1)	NA	
West South Central	42 (5.8)	25 143 (8.1)	NA	
East South Central	20 (2.8)	22 779 (7.3)	NA	
Mountain	16 (2.2)	13 775 (4.4)	NA	
Missing data	32 (4.4)	8578 (2.7)	NA	
Facility type				
Academic or research program	554 (76.3)	162 221 (52)	<.001	<.001
Integrated network cancer program	85 (11.7)	46 967 (15.1)	.02	
Comprehensive program	48 (6.6)	80 707 (25.9)	<.001	
Community program	7 (1.0)	13 549 (4.3)	<.001	
Missing data	32 (4.4)	8578 (2.7)	NA	
Zip code median income, US $				
<40 227	69 (9.5)	46 253 (14.8)	<.001	<.001
40 227-50 353	101 (13.9)	61 448 (19.7)	.002	
50 354-63 332	151 (20.8)	65 842 (21.1)	.41	
>63 333	258 (35.5)	95 093 (30.5)	<.001	
Missing data	147 (20.2)	43 386 (13.9)	NA	
Insurance status				
Private insurance	342 (47.1)	125 542 (40.2)	<.001	.001
Medicare	271 (37.3)	137 591 (44.1)	<.001	
Medicaid	68 (9.4)	26 509 (8.5)	.42	
Not insured	18 (2.5)	10 152 (3.3)	.29	
Other governmental insurance	10 (1.4)	6113 (2.0)	.33	
Unknown	17 (2.3)	6115 (2.0)	NA	
Charlson-Deyo comorbidity index^g^				
0	536 (73.8)	233 173 (74.7)	.61	.65
1	134 (18.5)	54 072 (17.3)	.45	
2	31 (4.3)	15 327 (4.9)	.48	
3	25 (3.4)	9450 (3.0)	.59	
p16 status (2018 or later)				
Positive	77 (15.3)	19 732 (19.9)	NA	.43
Negative	20 (4.0)	4057 (4.1)	NA	
Unknown	407 (80.8)	75 168 (76.0)	NA	
Combined stage				
I	90 (12.4)	94 786 (30.4)	<.001	<.001
II	78 (10.7)	40 517 (13.0)	.01	
III	125 (17.2)	37 858 (12.1)	.001	
IV	394 (54.3)	100 565 (32.2)	<.001	
NA	14 (1.9)	4002 (1.3)	NA	
Unknown	21 (2.9)	19 942 (6.4)	NA	

Abbreviation: NA, not applicable.

^a^ Unless otherwise indicated.

^b^ *P* value obtained from χ^2^ test of individual level (eg, for race comparisons made between White and races other than White), excluding unknown and missing data.

^c^ *P* value obtained from χ^2^ test of all levels, excluding unknown and missing data.

^d^ Area of residence was defined using the typology published by the United States Department of Agriculture Economic Research Service. Metropolitan counties were defined as metro areas with populations ranging from fewer than 250 000 to more than 1 million; urban counties had urban populations between 2500 and 20 000 or more, further classified by whether they were adjacent to a metro area; rural counties were completely rural or had fewer than 2500 urban residents, further classified by whether they were adjacent to a metro area.

^e^ Includes Chinese, Japanese, Filipino, Vietnamese, Thai, Loatian, Hmong, Asian Indian, and other Asian.

^f^ Other includes all groups besides Asian, Black, and White.

^g^ Charlson-Deyo comorbidity index range: 0, no; 1, mild; 2, moderate; and 3, severe comorbidities.

**Figure 1.**
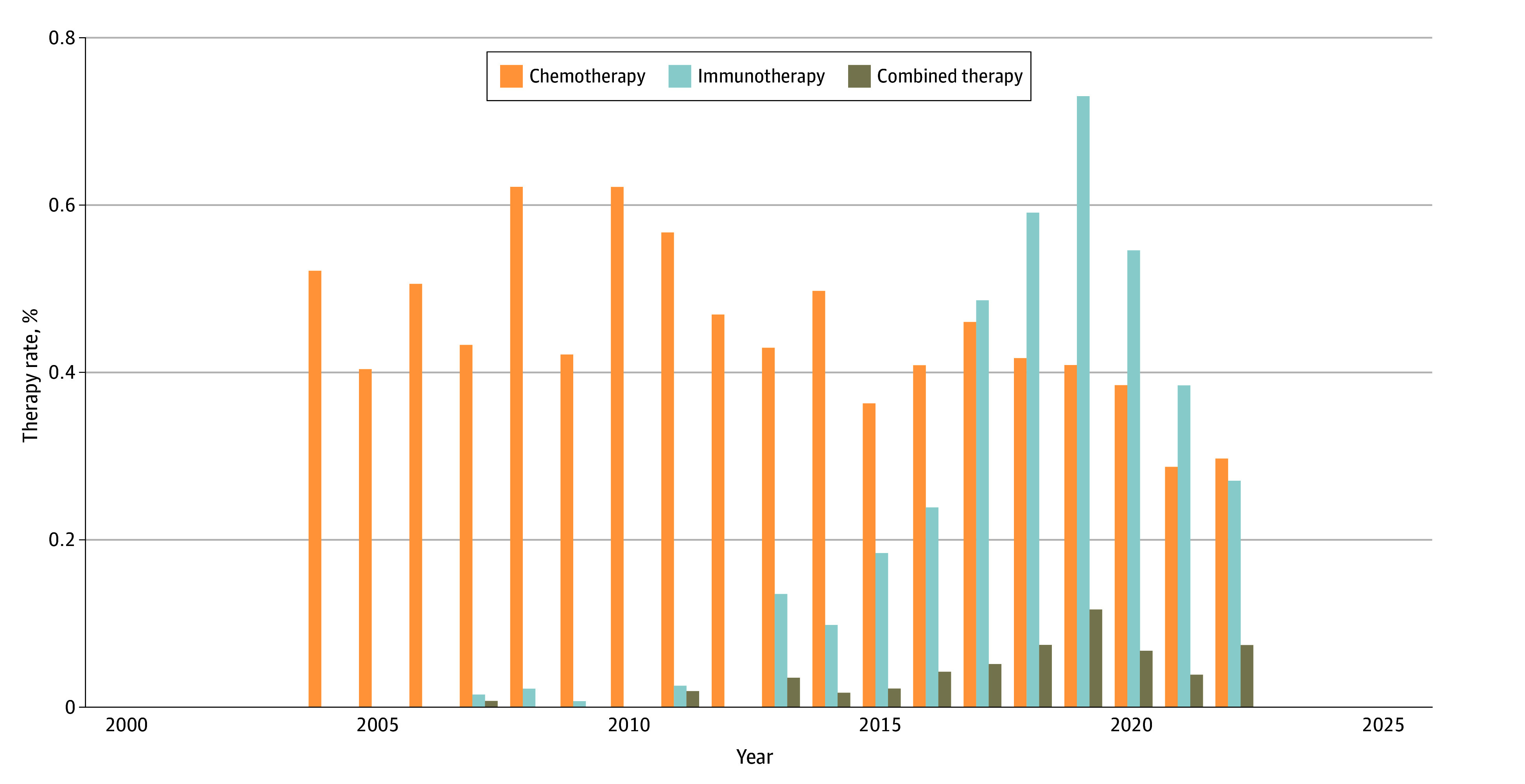
Absolute Rates for the Use of Neoadjuvant Chemotherapy, Immunotherapy, and Combined Chemotherapy-Immunotherapy From 2004 to 2022

From 2004 to 2022, there was a significant overall change in the rate of NST use (0.54% vs 0.47%; *P* < .001). Use was 0.54% in 2004, peaked at 1.02% in 2019, and ended at 0.47% in 2022. During this period, neoadjuvant chemotherapy use significantly decreased (0.52% to 0.30%; *P* < .001), whereas neoadjuvant immunotherapy use significantly increased (0.0% vs 0.27%; *P* < .001), with first use recorded in 2007 (0.02%). Annual use of neoadjuvant immunotherapy began in 2013 (0.14%) and peaked at 0.73% in 2019 (Figure 1). Sites with the largest increases in neoadjuvant immunotherapy use since 2013 were the hypopharynx (from 0.25% to 1.30%), gums and other oral cavity (from 0.19% to 0.58%), and tongue (from 0.18% to 0.27%).

**Figure 2.**
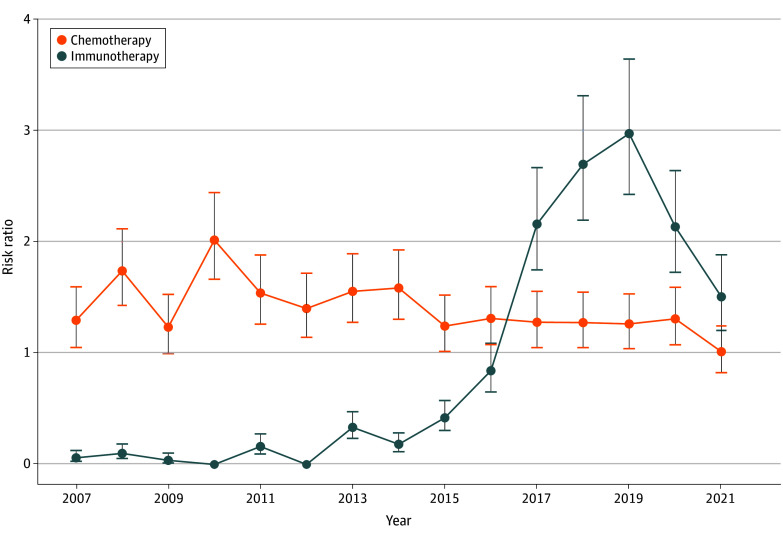
Adjusted Risk Ratios of Receiving Neoadjuvant Chemotherapy or Neoadjuvant Immunotherapy for Each Year of Diagnosis Compared With the Reference Year (2022) Error bars represent SEs. Year of diagnosis was used as a categorical variable in Poisson regression.

To understand the overall trend of neoadjuvant therapy use and the probability of any patient receiving this treatment paradigm, we performed a mixed-effects Poisson regression using reporting facility as the clustering variable. We obtained an intraclass correlation coefficient of 0.43 (95% CI, 0.22-0.67), indicating a strong grouping effect. After adjusting for demographic and clinical covariates, the probability of receiving neoadjuvant immunotherapy increased by 22.5% per year from 2007 to 2022 (RR, 1.22; 95% CI, 1.19-1.26). Restricting the analysis to pre–COVID-19 years (2007-2019), this increase was 55.2% annually (OR, 1.55; 95% CI, 1.47-1.63). In contrast, from 2007 to 2022 the probability of receiving neoadjuvant chemotherapy decreased by −2.5% per year (OR, 0.97; 95% CI, 0.96-0.99) (Figure 2).

## Discussion

This study, to our knowledge, provides the largest nationwide analysis of trends in NST use in surgically treated HNSCC. From 2004 to 2022, absolute rates of neoadjuvant therapy were modest, but our findings demonstrated a shift in practice patterns. The use of neoadjuvant immunotherapy has steadily increased, whereas the use of neoadjuvant chemotherapy has decreased.

The increase in neoadjuvant immunotherapy use coincided with increasing interest in ICIs as part of curative-intent treatment regimens. Early success of programmed cell death 1 inhibitors in recurrent or metastatic HNSCC, demonstrated in trials such as KEYNOTE-048 and CheckMate 141, established the efficacy of ICIs in advanced disease and paved the way for their investigation in earlier stages of treatment.^4,6^ Subsequently, multiple studies^8^ have evaluated the role of neoadjuvant immunotherapy. Most notably, the KEYNOTE-689 trial has further accelerated interest in incorporating immunotherapy into multimodal curative-intent treatment strategies.^7^

Our analysis showed that neoadjuvant immunotherapy use began to increase meaningfully around 2013, coinciding with the start of recruitment for KEYNOTE-012, which evaluated ICIs in the recurrent and metastatic disease setting.^5^ It is likely that this early increase in neoadjuvant immunotherapy represented increased off-label use. In 2015, accrual began for a wave of clinical trials evaluating ICIs in the neoadjuvant setting, which may explain the steep increase in ORs after 2015.^8^ Overall, adjusted risk of receiving neoadjuvant immunotherapy increased by a fourth annually between 2007 and 2022 and doubled annually before the COVID-19 pandemic. This upward trend reflects an increasing body of early-phase clinical trial evidence, increasing off-label use, and broader institutional adoption in academic settings.^8,9^

However, a noticeable decrease in the rate and risk of immunotherapy use occurred between 2020 and 2022. This decrease coincided with the COVID-19 pandemic, which caused substantial disruptions in cancer care globally. Several potential factors may explain this decrease, including delays in surgical scheduling, reductions in in-person clinical trial enrollment, and clinician hesitancy to use immunomodulatory agents in the setting of a global viral pandemic.^10^

Importantly, our mixed-effects Poisson regression revealed that the reporting facility accounted for nearly half of the variability in neoadjuvant therapy use, likely reflecting disparities between academic and nonacademic centers. Academic hospitals, where most neoadjuvant immunotherapy cases in our cohort were treated, were more likely to offer clinical trials and adopt novel approaches early. Community and nonacademic institutions may have lagged due to differences in resource availability and access to trials.^11^

Sociodemographic factors were also associated with the receipt of neoadjuvant immunotherapy. Recipients were more likely to be privately insured, reside in higher-income areas, and be treated at academic institutions and less likely to be of Black race. Our results are in line with reports of racial disparity in cancer care, such as the underrepresentation of Black and Latinx patients in oncology clinical trials.^12,13^ Our observations highlight the need to ensure equitable access to cutting-edge treatments as immunotherapy becomes integrated into standard of care for HNSCC.

### Limitations

This study has some limitations. The study is limited by its retrospective and observational nature. There is risk of selection bias because patients included in the NCDB may be systematically different from the overall population of patients with HNSCC. However, by collecting data on approximately 72% of incident cancer cases in the US, the NCDB covers a large proportion of the population.^14^ Another limitation is the inconsistent reporting of p16 and human papillomavirus status in the NCDB, which influenced the decision to exclude this variable as a fixed effect in our model. Notably, most trials evaluating neoadjuvant immunotherapy in HNSCC have not excluded patients based on p16 or human papillomavirus status.^8^

## Conclusions

In this retrospective cohort study of HNSCC, we highlight the increasing adoption of neoadjuvant immunotherapy in HNSCC, mirroring broader shifts in oncologic practices. Although the COVID-19 pandemic may have temporarily slowed this trend, emerging clinical trials are likely to inform an evolving standard of care for immunotherapy in the curative-intent primary treatment setting. Efforts should be made to address institutional and socioeconomic disparities in access to novel therapies to ensure all eligible patients can benefit from advances in treatment.

## Notes

Supplement.
Data Sharing Statement
