# Error in Funding/Support

**DOI:** 10.1001/jamanetworkopen.2025.49886

**Published:** 2025-12-01

In the Original Investigation titled “Trends in the Use of Neoadjuvant Systemic Therapy for Head and Neck Squamous Cell Carcinoma,”^1^ published October 28, 2025, the Funding/Support section was omitted in error. This article has been corrected.^1^
