# Supplementary material for: Trends in the Use of Neoadjuvant Systemic Therapy for Head and Neck Squamous Cell Carcinoma
Source: JAMA Netw Open. 2025 Oct 28;8(10):e2539778. doi: 10.1001/jamanetworkopen.2025.39778 (PMC12569713; doi:10.1001/jamanetworkopen.2025.39778)
Supplement: Supplement. — Data Sharing Statement [file jamanetwopen-e2539778-s001.pdf]

Data Sharing Statement

Cai. Trends in the Use of Neoadjuvant Systemic Therapy for Head and Neck Squamous Cell Carcinoma. *JAMA Netw Open*. Published October 28, 2025. doi:10.1001/jamanetworkopen.2025.39778

Data
Data available: No

Additional Information
Explanation for why data not available: Data is publicly available on the NCDB website
